# Frailty mediated the association between tooth loss and mortality in the oldest old individuals: a cohort study

**DOI:** 10.3389/fpubh.2023.1285226

**Published:** 2024-01-24

**Authors:** MingXia Wang, Xiaomeng Deng, Hanjie Chen, Yuhan Diao, Chang Liu, Jun Gao, Xin Tang, Xiaoyan Li, Yan Liu, Jun Duan

**Affiliations:** ^1^Department of Stomatology, Shenzhen Luohu Hospital of Traditional Chinese Medicine, Shenzhen Hospital of Shanghai University of Traditional Chinese Medicine, Shenzhen, China; ^2^Department of Comprehensive Ward, Peking University Shenzhen Hospital, Shenzhen, China; ^3^Department of Medical Record Statistics, Peking University Shenzhen Hospital, Shenzhen, China; ^4^Department of Stomatology, Peking University Shenzhen Hospital, Shenzhen, China

**Keywords:** cohort studies, inflammation, nutrition, oral medicine, public health

## Abstract

**Introduction:**

Tooth loss is associated with increased mortality risk; however, the mechanism underlying this is still not clear. The objective of this study was to explore whether frailty mediates the association between tooth loss and mortality risk among the oldest old individuals.

**Methods:**

The participants were followed up from 1998 to 2018 in the Chinese Longitudinal Healthy Longevity Survey (CLHLS). Frailty was constructed following a standard procedure. Mortality, frailty, and tooth loss were applied as the outcome, mediator, and independent variables, respectively. The Cox model was fitted, including possible confounders, for causal mediation analysis. A total effect (TE), an average causal mediation effect (ACME), an average direct effect (ADE), and a proportion mediated (PM) effect were calculated.

**Results:**

During the 129,936 person-years at risk, 31,899 individuals with a mean age of 91.79  years were included. The TE and ADE of severe tooth loss on mortality were 0.12 (95% CI: 0.08, 0.15) and 0.09 (95% CI: 0.05, 0.13); the ACME of frailty was 0.03 (95% CI: 0.02, 0.03) with 21.56% of the TE being mediated.

**Discussion:**

This study illustrated that tooth loss is associated with mortality, and frailty appeared to mediate the relationship. It is recommended that oral health indicators and frailty status be incorporated into routine geriatric assessments to promote optimal oral health and non-frailty status.

## Introduction

Tooth loss is identified as the primary indicator of poor oral health, which is identified as the primary indicator of healthy aging (1). Over the past decade, considerable research efforts have been devoted to the finding that tooth loss is associated with an increased risk of mortality (2–5). However, the mediators of the association between tooth loss and mortality remain poorly reported, especially in the oldest old individuals.

Current studies suggest that malnutrition and inflammation are thought to be the main paths between tooth loss and increased mortality risk (6–11). However, the majority of prior research on the aforementioned mechanisms remains speculative, with only a limited number of studies having undergone rigorous validation. In our current review, we have identified a single study within the Japanese cohort (*N* = 891) that employs mediation analysis to examine the relationship between nutritional status, tooth loss, and mortality. Interestingly, this study reveals a significant association between nutritional status and both tooth loss and mortality, while systemic inflammation does not exhibit such a correlation (12). Additionally, another study highlights weight loss as a clinically significant indicator of malnutrition. Through the utilization of follow-up mail self-reported questionnaires, it was determined that weight loss serves as a mediator in the link between tooth loss and mortality among older adults (13). However, the proportion of the above mediating effects (13.10%) that can be explained warrants further exploration of other mediating factors. In addition, no further subgroup analyses were performed in the previous studies. On the whole, limited data representations of older populations and appropriate mediators may be responsible for the lack of research.

Frailty is considered a major public health problem because of its high prevalence in the older adults population (14, 15). It was reported to be a more important predictor of mortality than biological age (16). There were quite a few studies that also reported that tooth loss could lead to frailty (17–19). Moreover, in the association of tooth loss-frailty and frailty–mortality, there were significant associations with both malnutrition and inflammation (20–23). As described, we put forward the hypothesis that tooth loss could be associated with mortality mediated by frailty.

Therefore, in this study, a large prospective cohort study in China examined the relationship between tooth loss and mortality and emphatically examined how frailty mediates the relationship between tooth loss and mortality among the oldest old individuals.

## Method

### Study design and participants

**Figure 1:**
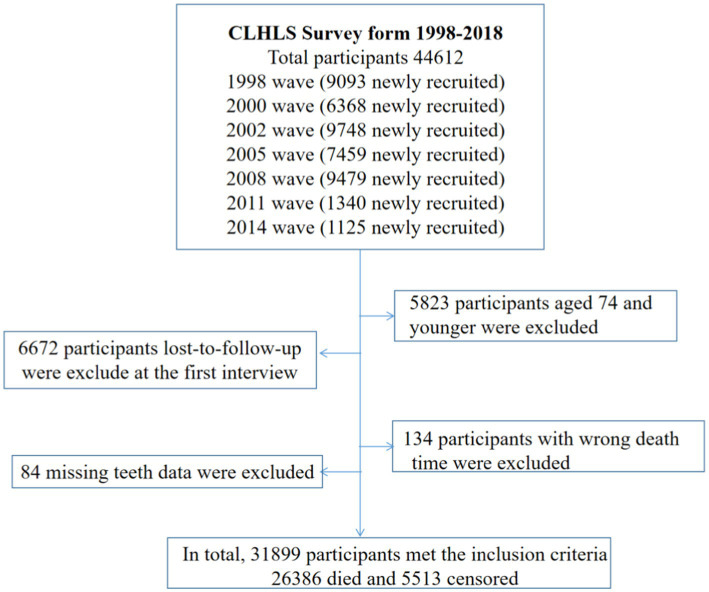
Cohort selection criteria, Chinese Longitudinal Healthy Longevity Study (CLHLS), surveyed from 1998 to 2018.

The Chinese Longitudinal Healthy Longevity Survey (CLHLS) was used, which is the most comprehensive survey of the oldest old individuals in China. This research aimed to gain a better understanding of the healthy longevity of human societies and to determine which biological, behavioral, social, and traditional environmental risk factors play an important role. It began in 1998 with a random selection of half of the cities and counties in 23 provinces of China, followed by follow-ups in 2000, 2002, 2005, 2008, 2011, 2014, and 2018. Detailed information about the CLHLS can be found elsewhere (24, 25). The baseline examinations were conducted using a standardized and objective method, administered by research staff who had undergone rigorous training and assessment. The field investigation team was composed of three investigators, including a leader (responsible for the coordination and organization of the team and the quality control of the investigation), an interviewer (responsible for interviews and questionnaires), and a physician (responsible for physical examination and collection of blood and urine samples). The team members collaborated closely with each other, ensuring a cohesive approach. In this study, a total of 44,612 older adults were included at baseline. We excluded 5,823 older adults aged 74 and younger, 6,672 older adults were lost to follow-up at the first follow-up survey, 134 older adults had incorrect death time, and 84 older adults had missing teeth data (Figure 1). A more detailed design of the CLHLS is described in Appendix 1.

### Outcome variable

After the follow-up survey, the main outcome was mortality from all causes. The survival status of the older adults was determined by family members or relatives, determining whether or not the older adults completed the survey, died, were lost to follow-up, or were missing from the study. Lost follow-up was ascertained when older adults were not found when contacted. Censored older adults are those who survived but were lost to follow-up.

### Explanatory variables

The number of remaining teeth at baseline was mainly used to assess tooth loss status. To determine the status of tooth loss, the following question was asked: “Can you tell me how many natural teeth you still have?” Based on the remaining number of natural teeth, we divided them into four categories: 0, 1–9, 10–19, and 20+.

### Mediators

Frailty was constructed using a standard method. The component of frailty was comprised of deficits associated with health status if the following criteria were met: Multi-system and physiological deficits were involved. In accordance with evidence-based research (14, 16), an older adult’s frailty was calculated by dividing their number of deficits by the number of deficits they have overall. There were 30 indicators of health deficits that included eight major sets of components following the established research methodology: chronic disease conditions (self-reports from a list of 11 diseases), cognitive functioning, activity of daily living disability (needing help in performing the 6 basic daily activities), functional limitations (5 objective examinations of physical function), self-rated health, visual and auditory functions, psychological distress, and others (e.g., rhythm of the heart, interviewer-rated health, number of serious illnesses in the past 2 years). Appendix Table 1 provides details of all items. The definitions of some indicators are explained in Appendix 2. Frailty items are similar to those of studies conducted in China (14, 16), Canada (26), and the United States (27).

In each deficit, the 0–1 interval was dichotomized. The healthiest state is 0 (absence of a deficit), whereas the unhealthiest state is 1 (maximal deficit expression). Finally, the total score of all questions is divided by the total value of 30 points. The resulting value is the frailty index of the respondent, which ranges from 0 to 1. The higher the score, the higher the frailty.

### Covariates

As covariates, the possible confounders were evaluated based on clinical knowledge and previous studies (3, 4). The factors included sex, age, education, marital status, smoking status, drinking status, dietary diversity, and leisure activities such as reading, watching TV, listening to the radio, doing housework, keeping pets, and growing flowers. Education background was categorized as “literate” if a participant had received >1 year of any formal education and “illiterate” if a participant had not received formal education. Dietary diversity was assessed by nine major food groups (meat, fish and seafood, eggs, beans, fruits, vegetables, tea, garlic, and sugar or candy), which were recorded as “often or almost every day” or “occasionally” or “rarely or never.” One point of dietary diversity was defined as often or almost every day consuming any food group without considering a minimum intake; the maximum possible dietary diversity was 9 points (25). Leisure activities were divided into three categories (never, sometimes, and often). More details are described in Appendix 2.

### Statistical analysis

Among the covariates, less than 1.09% of values were missing, and mean value imputation was applied. Age variables were summarized using interquartile ranges, and categorical variables were summarized using frequency and percentage. For categorical variables, the Cochran–Mantel–Haenszel test was used, and for continuous variables, the Kruskal–Wallis test was used.

**Figure 2:**
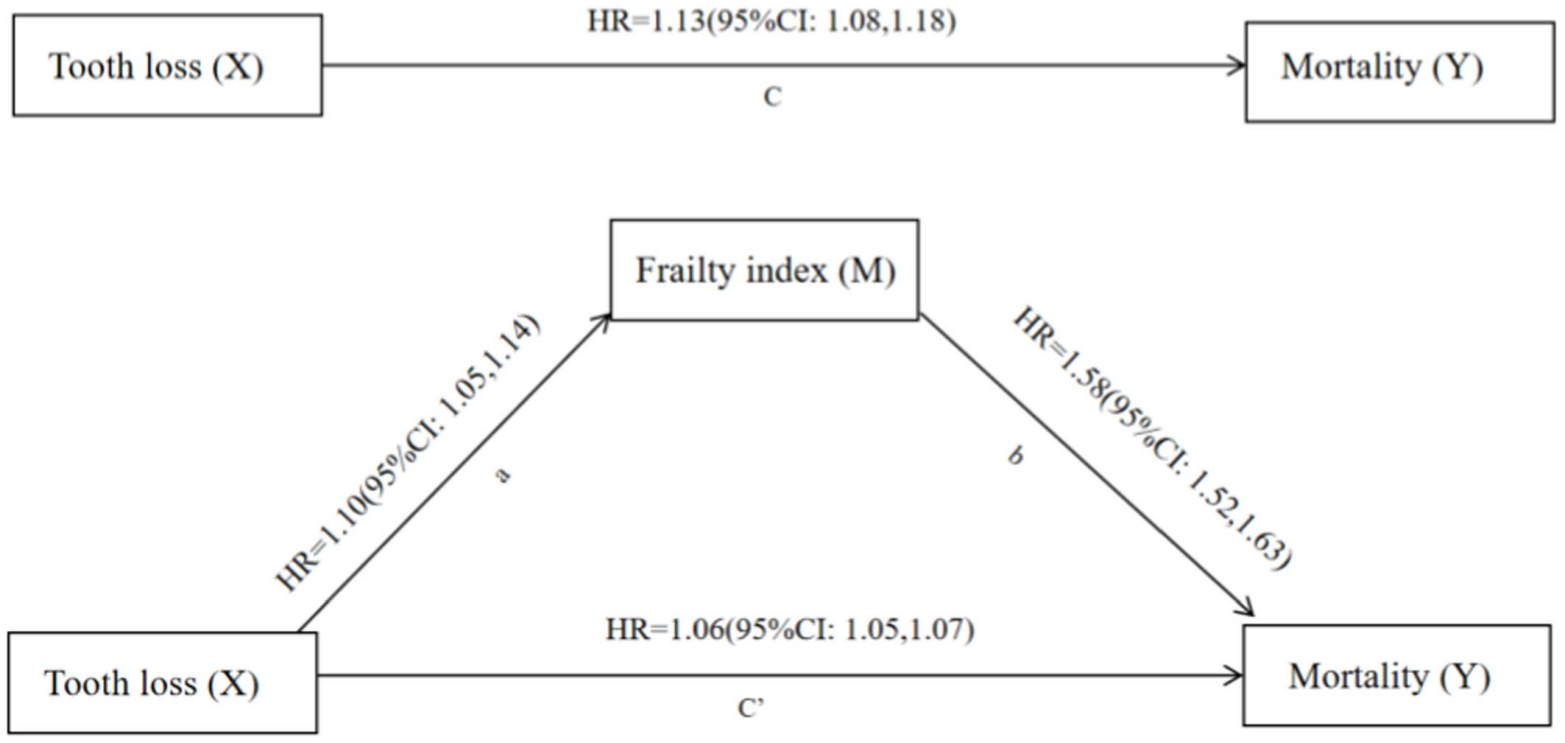
Frailty index mediates the relationship between tooth loss and mortality. X, independent variable (cause); Y, dependent variable (outcome); M, mediator; Path a, the relationship between X and M; Path b, the relationship between M and Y, with X included in the model; Path c, the relationship between X and Y; Path c’, the relationship between X and Y, with M included in the model; HR, odds ratio; all models were adjusted for sex, age, education, marital status, smoking status, drinking status, dietary diversity, and leisure activities including doing housework, reading, watching TV, listening to the radio, keeping pets, and growing flowers.

The causal mediation analysis was conducted based on the framework for potential outcomes to investigate the mediating effects of frailty on tooth loss and mortality risk. This diagram in Figure 2 illustrates the hypothesized causal chain. In this study, the independent variable was proposed as X (tooth loss), the dependent variable was proposed as Y (subsequent mortality), and the mediator was proposed as M (frailty). A total effect (TE), an average causal mediation effect (ACME), an average direct effect (ADE), and a proportion mediated (PM) effect were estimated. The first step is to fit a mediator model in which tooth loss is modeled as a function of frailty comorbid with covariates (sex, age, education, marital status, smoking status, drinking status, dietary diversity, and leisure activities including reading, watching TV, listening to the radio, doing housework, keeping pets, and growing flowers). The next step is to model the survival variables, which are the outcome variables, including tooth loss, frailty, and covariates. As in the mediator model, the outcome model includes an explanation for tooth loss and mediation for frailty. We use the COX model for the mediator and outcome models, respectively (28).

The potential outcomes framework was to define these quantities. Let *M_i_* (*t*) denote the potential value of a mediator of interest for unit *i* under the treatment status *T_i_* = *t*. Let *Y_i_*(*t, m*) denote the potential outcome that would result if the treatment and mediating variables equal *t* and *m*, respectively. Consider a standard experimental design where only the treatment variable is randomized. We observe only one of the potential outcomes, and the observed outcome, *Y_i_*, equals *Y*_i_(*T_i_, M_i_*(*T_i_*)) where M_i_(*T_i_*) represents the observed value of the mediator *M_i_*. With this notation, the total unit treatment effect can be written as,

$T_{i}\text{≡}Y_{i}\left(1,M_{i}\left(1\right)\right)-Y_{i}\left(0,M_{i}\left(0\right)\right)$ (1)

We can decompose this total effect into two components (29). First, the causal mediation effects are represented (30).

$\delta_{i}\left(t\right)=Y_{i}\left(t,M_{i}\left(1\right)\right)-Y_{i}\left(t,M_{i}\left(0\right)\right)$ (2)

for each treatment status, *t* = 0, 1. All other causal mechanisms can be represented by the direct effects of the treatment as follows (28),

$\zeta i\left(t\right)\text{≡}Y_{i}\left(1,M_{i}\left(t\right)\right)-Y_{i}\left(0,Mi\left(t\right)\right)$ (3)

for each unit *i* and each treatment status, *t* = 0, 1. Together, we see that they sum up to the total effect,

$T_{i}=\delta_{i}\left(t\right)+\zeta_{i}\left(1-t\right)$ (4)

for *t* = 0, 1. The average causal mediation effects (ACMEs) $\bar{\delta }$ (*t*) and the average direct effects (ADEs) $\bar{\zeta }$ (*t*) represent the population averages of these causal mediation and direct effects (31).

To check the robustness of the primary results, we excluded missing data for sensitivity analysis. In addition, this study has been adjusted to exclude mortality in the first 0.5 and 1 years due to the possibility that the increase in frailty before mortality in the last year of life could influence the results.

All data were analyzed using R 4.2.2 with the packages “mediation” and “survival.” Statistical significance was determined by an alpha value of 0.05 (two-sided).

### Ethics approval

The Protection of Human Subjects for the CLHLS was approved by the biomedical ethics committee of Peking University (IRB00001052-13074). Informed and written consent were obtained from all participants and/or their relatives. In addition, a STROBE statement was followed to assess our study in Appendix 3.

## Results

During the 129,936 person-years at risk, we included 31,899 oldest old individuals with a mean age of 91.79 years old. The baseline characteristics of the oldest old individuals are presented in Table 1. When more teeth were missing, age and frailty scores increased, ranging from 84 to 95 years old and 0.09 to 0.14 points, respectively. When more teeth were missing, some covariates accounted for an increasing proportion: the proportion of deaths increased from 71.66 to 85.20%; the proportion of illiteracy increased from 53.58 to 75.50%; and the proportion of the lowest dietary diversity score increased from 20.71 to 26.72%. When more teeth were missing, some covariates accounted for a decreasing proportion: the proportion of exercising regularly decreased from 35.65 to 21.75%; the proportion of doing housework decreased from 40.77 to 22.33%; the proportion of playing cards decreased from 7.17 to 3.03%; and the proportion of watching TV or listening to the radio decreased from 46.28 to 27.87%.

**Table 1:** Descriptive characteristics of the participants at baseline.

Variables	Total (*n* = 31,899)	>20 teeth (*n* = 3,027)	10–19 teeth (*n* = 4,036)	1–9 teeth (*n* = 11,563)	Anodontia (*n* = 13,273)	*p*
Age, Median (Q1, Q3)	92 (85, 100)	84 (80, 91)	87 (81, 93)	92 (86, 100)	95 (89, 101)	<0.01
Sex, *n* (%)						<0.01
Male	19,432 (60.92)	1,315 (43.44)	2,038 (50.50)	6,913 (59.79)	9,166 (69.06)	
Female	12,467 (39.08)	1,712 (56.56)	1,998 (49.50)	4,650 (40.21)	4,107 (30.94)	
Survival state, *n* (%)						<0.01
Survival	5,513 (17.28)	858 (28.34)	884 (21.90)	1,807 (15.63)	1,964 (14.80)	
Death	26,386 (82.72)	2,169 (71.66)	3,152 (78.10)	9,756 (84.37)	11,309 (85.20)	
Frailty index, Median (Q1, Q3)	0.12 (0.07, 0.19)	0.09 (0.05, 0.15)	0.10 (0.06, 0.16)	0.12 (0.07, 0.18)	0.14 (0.09, 0.22)	<0.01
Education, *n* (%)						<0.01
Non-illiteracy	9,581 (30.04)	1,405 (46.42)	1,571 (38.92)	3,353 (29.00)	3,252 (24.50)	
Illiteracy	22,318 (69.96)	1,622 (53.58)	2,465 (61.08)	8,210 (71.00)	10,021 (75.50)	
Marital status, *n* (%)						<0.01
Yes	26,463 (82.96)	2,480 (81.93)	3,206 (79.44)	9,502 (82.18)	11,275 (84.95)	
No	5,436 (17.04)	547 (18.07)	830 (20.56)	2,061 (17.82)	1,998 (15.05)	
Dietary diversity, *n* (%)						<0.01
Score = 6	2,673 (8.38)	414 (13.68)	356 (8.82)	815 (7.05)	1,088 (8.20)	
Score = 5	2,740 (8.59)	327 (10.80)	393 (9.74)	928 (8.03)	1,092 (8.23)	
Score = 4	4,321 (13.55)	453 (14.97)	551 (13.65)	1,522 (13.16)	1,795 (13.52)	
Score = 3	6,299 (19.75)	607 (20.05)	782 (19.38)	2,281 (19.73)	2,629 (19.81)	
Score = 2	7,381 (23.14)	599 (19.79)	938 (23.24)	2,722 (23.54)	3,122 (23.52)	
Score = 1	8,485 (26.60)	627 (20.71)	1,016 (25.17)	3,295 (28.50)	3,547 (26.72)	
Smoke, *n* (%)						<0.01
No	22,490 (70.50)	1,904 (62.90)	2,682 (66.45)	8,142 (70.41)	9,762 (73.55)	
Yes	9,409 (29.50)	1,123 (37.10)	1,354 (33.55)	3,421 (29.59)	3,511 (26.45)	
Drink, *n* (%)						<0.01
No	22,856 (71.65)	1,946 (64.29)	2,808 (69.57)	8,143 (70.42)	9,959 (75.03)	
Yes	9,043 (28.35)	1,081 (35.71)	1,228 (30.43)	3,420 (29.58)	3,314 (24.97)	
Exercise regularly, *n* (%)						<0.01
0	23,956 (75.10)	1,948 (64.35)	2,803 (69.45)	8,819 (76.27)	10,386 (78.25)	
1	7,943 (24.90)	1,079 (35.65)	1,233 (30.55)	2,744 (23.73)	2,887 (21.75)	
Garden work, *n* (%)						<0.01
Often	1,641 (5.14)	311 (10.27)	275 (6.81)	501 (4.33)	554 (4.17)	
Sometimes	1,391 (4.36)	207 (6.84)	262 (6.49)	484 (4.19)	438 (3.30)	
Never	28,867 (90.49)	2,509 (82.89)	3,499 (86.69)	10,578 (91.48)	12,281 (92.53)	
Housework, *n* (%)						<0.01
Often	9,040 (28.34)	1,232 (40.70)	1,540 (38.16)	3,304 (28.57)	2,964 (22.33)	
Sometimes	4,927 (15.45)	550 (18.17)	717 (17.77)	1,890 (16.35)	1,770 (13.34)	
Never	17,932 (56.21)	1,245 (41.13)	1,779 (44.08)	6,369 (55.08)	8,539 (64.33)	
Play card, *n* (%)						<0.01
Often	9,040 (28.34)	1,232 (40.70)	1,540 (38.16)	3,304 (28.57)	2,964 (22.33)	
Sometimes	4,927 (15.45)	550 (18.17)	717 (17.77)	1,890 (16.35)	1,770 (13.34)	
Never	17,932 (56.21)	1,245 (41.13)	1,779 (44.08)	6,369 (55.08)	8,539 (64.33)	
Watch TV or listen to the radio, *n* (%)						<0.01
Often	9,837 (30.84)	1,401 (46.28)	1,547 (38.33)	3,190 (27.59)	3,699 (27.87)	
Sometimes	7,790 (24.42)	759 (25.07)	1,062 (26.31)	3,004 (25.98)	2,965 (22.34)	
Never	14,272 (44.74)	867 (28.64)	1,427 (35.36)	5,369 (46.43)	6,609 (49.79)	

Figure 2 shows that tooth loss and mortality were mediated based on the causal steps method. For path a, tooth loss was associated with a frailty hazard ratio (HR) of 1.10 (95% confidence interval (CI): 1.05, 1.14). Based on path b, mortality was associated with frailty of 1.58 (95%CI: 1.52, 1.63) after adjusting for all covariates and tooth loss. Based on path c, there was an association between tooth loss and mortality of 1.13 (95%CI: 1.08, 1.18). Adding frailty to the models (path c’) decreased the HR of tooth loss on mortality by 1.06 (95%CI: 1.05, 1.07), suggesting that frailty may mediate the relationship. Sensitivity analysis showed that the results remained stable in Appendix Figures 1–3.

**Figure 3:**
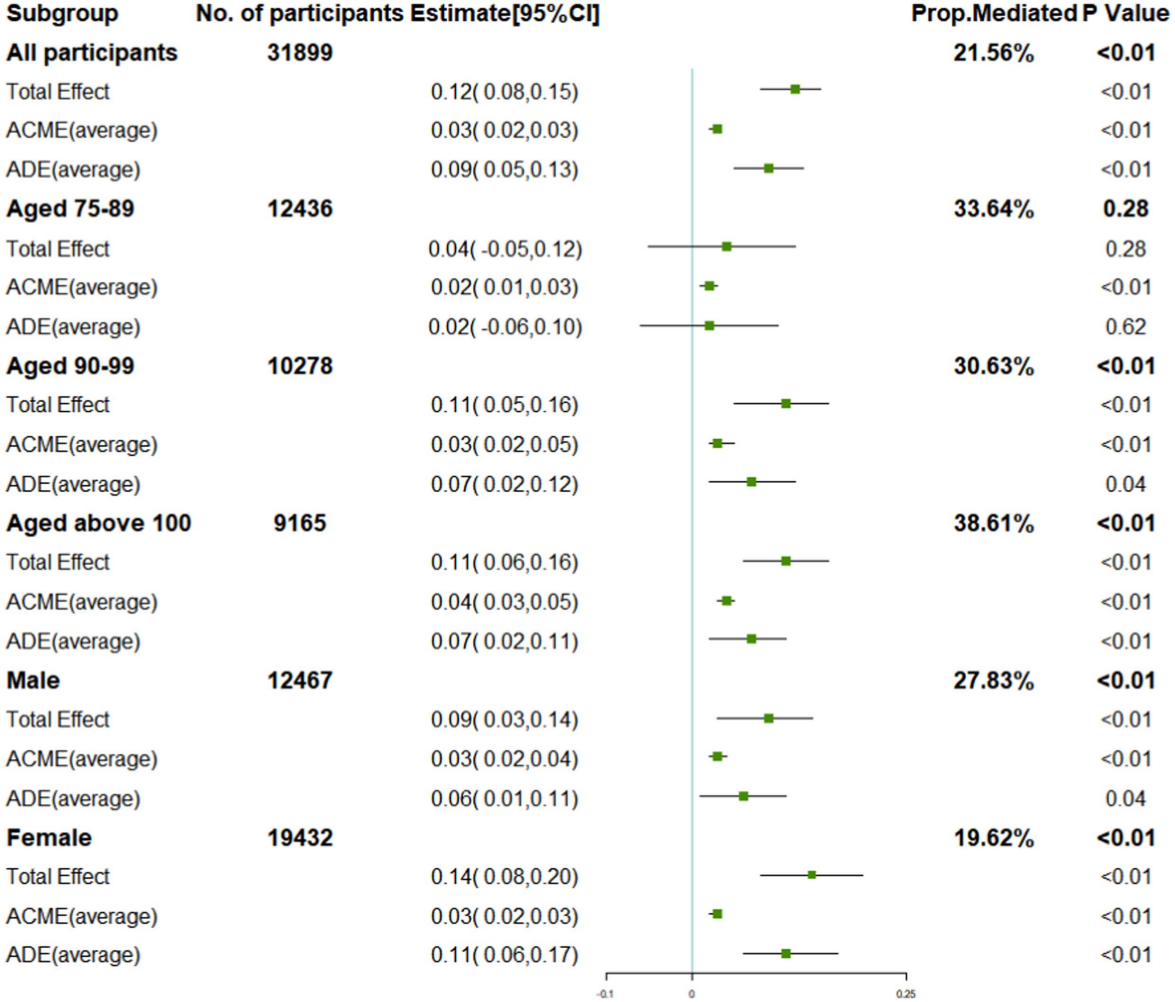
Direct and indirect effects of tooth loss and mediators on mortality and subgroup analysis.

Further analysis was conducted based on the above results to determine if tooth loss and death are mediated by frailty. In Figure 3, the TE and ADE of severe tooth loss on mortality were 0.12 (95%CI: 0.08, 0.15) and 0.09 (95 %CI: 0.05, 0.13); the ACME of frailty was 0.03 (95 %CI: 0.02, 0.03) with 21.56% (*p* < 0.01) of the total effect being mediated. In the age subgroup, as the age group increased, the ACME of frailty was 0.02 (95 %CI: 0.01, 0.03) with 33.64% (*p* = 0.28) of the TE being mediated, 0.03 (95 %CI: 0.02, 0.05) with 30.63% (*p* < 0.01) of the TE being mediated, and 0.04 (95 %CI: 0.03, 0.05) with 38.61% (*p* < 0.01) of the TE being mediated, respectively. In the sex subgroup, the ACME of frailty was 0.03 (95 %CI: 0.02, 0.04) with 27.38% (*p* < 0.01) of the TE being mediated in the male and 0.03 (95 %CI: 0.02, 0.03) with 19.62% (*p* < 0.01) of the TE being mediated in the female.

## Discussion

A primary finding of this prospective cohort with a mean age of 91.79 years old is that tooth loss is mediated by frailty and represents 21.56% of the TE. In the subgroup analysis, frailty played a more important role in mediating the association between tooth loss and mortality in males (27.83%) than in females (19.62%), and the mediation proportion increased with age from 30.63 to 38.61%.

The present study adds to the limited amount of research exploring frailty as a mediator of tooth loss and mortality among the oldest old individuals. Until now, the mediators of tooth loss and mortality have been poorly understood. Most studies on malnutrition and inflammation mechanisms are still hypotheses. In the Japanese Tsurugaya project, mortality and tooth loss were mediated by nutritional status, while systemic inflammation was not (12). However, the large confidence intervals suggested inadequate power or a small sample size. Another Japan Gerontological Evaluation Study suggested that mortality increases with tooth loss that is mediated by weight loss (13). However, this study was followed up by self-reported questionnaires, and the proportion of the above mediating effects (13.10%) that can be explained warrants further exploration of other mediating factors. In addition, no further subgroup analyses were performed in the previous studies.

In this study, our work explored frailty as a mediator, which could reflect both inflammation and malnutrition (32, 33). Frailty has been implicated as a mediator in the association between tooth loss and high mortality in the following possible ways: Tooth loss might trigger higher levels of inflammation (34), which can lead to frailty and increase mortality risk. Alternatively, tooth loss can cause a change in food selection and nutrient intake that leads to malnutrition as well as frailty and increased mortality risk (35).

In the subgroup analysis, men were more affected by frailty than women when it came to the relationship between tooth loss and mortality. In general, the health-survival paradox describes how men live shorter lives but have fewer disabilities than women (36). Frailty may be more prevalent in women due to higher incidence, longer duration (i.e., low recovery), and lower severity of illness (37). Basically, a sudden death in men due to frailty is more likely, whereas women experience gradual degeneration over time. It may be to be expected that men are associated with a higher proportion of mediating explanations according to frailty between tooth loss and mortality. Regarding the age group, it is common for participants to become frailer as they age. In most cases, frailty will worsen rather than improve over time, as it is a dynamic state (15). That may be why the proportion of mediating explanations increases with age. Overall, our results provide further evidence for the mechanisms underlying subgroup differences in the degree to which frailty mediates tooth loss and mortality.

From a public health and clinical standpoint, it is imperative to prioritize the preservation of non-frailty status in the oldest old population with tooth loss in order to mitigate subsequent mortality. Given the increasing prevalence of frailty and its association with tooth loss and mortality, the implications of frailty on the wellbeing of aging individuals and the strained healthcare system are significant. Considering the extent of tooth loss and frailty in patients can assist clinicians in delivering treatment that is more tailored to the individual’s needs. In turn, delaying tooth loss and frailty through primary, secondary, and tertiary prevention could lead to better outcomes. Even though we found that the relationship between tooth loss and mortality appears to be mediated by frailty, a more thorough evaluation of frailty assessment and targeted treatment is needed to determine whether they will benefit patients and healthcare systems.

In our study, we took advantage of a prospective design and included a population of the oldest old individuals as well as a wide range of covariates for a comprehensive analysis of the associations. There are, however, several limitations to consider. First and foremost, longitudinal studies face the long-term problem of loss of follow-up. The attrition rates in cohort studies of older adults individuals are inevitable. The second issue is the reporting bias in self-reported oral health data, despite several studies showing its reliability and validity (38, 39). Third, this study might benefit from trajectories of tooth loss rather than scores based on the baseline number of teeth. Further research and evaluation are needed in future. Finally, residual confounding may have impacted our findings, even after accounting for many possible covariates. Nevertheless, we hope that this study will facilitate a better understanding of tooth loss, frailty, and mortality.

## Conclusion and implications

In this study, tooth loss was associated with mortality, and frailty mediated the association. A key recommendation was the inclusion of oral health indicators and frailty status in routine geriatric assessments in order to maintain good oral health and non-frailty status in older adults. For future longitudinal studies to explore the association between tooth loss and mortality, frailty would be a better measure because it reflects a comprehensive geriatric status and may be more responsive to changes in nutrition and inflammation index.

## Data availability statement

The raw data supporting the conclusions of this article will be made available by the authors, without undue reservation.

## Ethics statement

The studies involving humans were approved by the Biomedical Ethics Committee of Peking University (IRB00001052-13074). The studies were conducted in accordance with the local legislation and institutional requirements. The participants provided their written informed consent to participate in this study.

## Author contributions

MW: Conceptualization, Data curation, Formal analysis, Visualization, Writing – original draft, Writing – review & editing. XD: Conceptualization, Formal analysis, Funding acquisition, Supervision, Writing – review & editing. HC: Formal analysis, Methodology, Supervision, Writing – review & editing. YD: Formal analysis, Investigation, Methodology, Project administration, Writing – review & editing. CL: Formal analysis, Methodology, Writing – review & editing. JG: Data curation, Methodology, Writing – review & editing. XT: Data curation, Methodology, Writing – review & editing. XL: Data curation, Methodology, Writing – review & editing. YL: Data curation, Methodology, Writing – review & editing. JD: Conceptualization, Funding acquisition, Methodology, Supervision, Writing – original draft, Writing – review & editing.
