# Supplementary material for: Frailty mediated the association between tooth loss and mortality in the oldest old individuals: a cohort study
Source: Front Public Health. 2024 Jan 24;11:1285226. doi: 10.3389/fpubh.2023.1285226 (PMC10848322; doi:10.3389/fpubh.2023.1285226)
Supplement: Supplementary file 1 [file Presentation_1.pdf]

Frailty mediated the association between tooth loss and mortality in the oldest old individuals

Appendices:

Appendix 1. Study design of CLHLS.

Appendix table 1. List of 30 variables included in the Frailty index.

Appendix 2. Definition of related covariates for CLHLS.

Appendix figure 1. Frailty index mediate the relationship between tooth loss and mortality after excluding missing data.

Appendix figure 2. Frailty index mediate the relationship between tooth loss and mortality after excluding mortality in the first 0.5 year.

Appendix figure 3. Frailty index mediate the relationship between tooth loss and mortality after excluding mortality in the first 1 year.

Appendix 3 STROBE Statement—checklist of items that should be included in reports of observational studies.

Appendix 1. Study design of CLHLS.

The CLHLS has the largest sample of centenarians in the world. Data quality, including assessments of mortality rate, proxy use, non-response rate, sample attrition, reliability and validity of major health measures, and the rates of logically inconsistent answers, have been conducted extensive evaluations with generally satisfactory results compared to other major aging studies (Zeng et al., 2017).

Until present, the CLHLS conducted face-to-face interviews in 1998, 2000, 2002, 2005, 2008-09, 2011-12, 2014 and 2018, respectively, using the instruments of the Danish longevity survey analyzed by Christensen and colleagues. The instruments were adapted to the Chinese culture and socioeconomic context. Since the 2002 wave, the CLHLS was expanded from only recruiting oldest-old in 1998 and 2000 waves to also interviewing approximately three randomly selected nearby elders predefined age and sex in conjunction with every two centenarians. The CLHLS adopted a targeted random-sample design to ensure representativeness, even distribution across age and gender and sufficient sub-sample size of the oldest-old aged 80+.

Zeng Y, Feng Q, Hesketh T, Christensen K, Vaupel JW. 2017. Survival, disabilities in activities of daily living, and physical and cognitive functioning among the oldest-old in china: a cohort study. Lancet. 389(10079): 1619-1629.

Appendix table 1. List of 30 variables included in the Frailty index.

No.	Definition according to baseline self-report, physical measurements, or both	Coding of variables
1	Self-reported diagnosis of hypertension	Yes=1.00;No=0.00
2	Self-reported diagnosis of diabetes	Yes=1.00;No=0.00
3	Self-reported diagnosis of tuberculosis	Yes=1.00;No=0.00
4	Self-reported diagnosis of heart disease	Yes=1.00;No=0.00
5	Self-reported diagnosis of stroke/cerebrovascular disease	Yes=1.00;No=0.00
6	Self-reported diagnosis of bronchitis, emphysema, asthma, or pneumonia	Yes=1.00;No=0.00
7	Self-reported diagnosis of cancer	Yes=1.00;No=0.00
8	Self-reported diagnosis of arthritis	Yes=1.00;No=0.00
9	Self-reported diagnosis of bedsores	Yes=1.00;No=0.00
10	Self-reported diagnosis of gastric or duodenal ulcers	Yes=1.00;No=0.00
11	Self-reported diagnosis of Parkinson's disease	Yes=1.00;No=0.00
12	Number of serious illnesses in the past 2 yearsa	Two or more illnesses are assigned a value of 1
13	Functional limitations: Unable to put hand behind neck	Both hand=1.00;sigle hand=0.5;Neither hand=0
14	Functional limitations: Unable to put hand behind lower back	Both hand=1.00;sigle hand=0.5;Neither hand=0
15	Functional limitations: Unable to stand up from sitting in a chair	Yes,without using hand=0.00;Yes,with using hand=0.50;No=1.00
16	Functional limitations: Unable to pick up a book from the floor	Yes,without using hand=0.00;Yes,with using hand=0.50;No=1.00
17	ADLs: Needs assistance bathing	Without assistance=0.00;One part assistance=0.50;More than one part assistance=1
18	ADLs: Needs assistance dressing	Without assistance=0.00;One part assistance=0.50;More than one part assistance=1
19	ADLs: Needs assistance toileting	Without assistance=0.00;One part assistance=0.50;More than one part assistance=1
21	ADLs: Needs assistance in indoor transferring	Without assistance=0.00;One part assistance=0.50;More than one part assistance=1
22	ADLs: Needs assistance eating	Without assistance=0.00;One part assistance=0.50;More than one part assistance=1

23	ADLs: Incontinence	Without assistance=0.00;One part assistance=0.50;More than one part assistance=1
24	Cognitively impaired (based on the Mini Mental State Examination)**See Table R1 for details**	Normal=0; Slightly impaired=0.50;severely impaired=1.00
25	Poor self-rated health	Poor=1.00; fair=0.50; good=0.25; excellent=0.00
26	Poor interviewer-rated health	Poor=1.00; fair=0.50; good=0.25; excellent=0.00
27	Hearing loss	Yes=1.00;No=0.00
28	Vision loss	Yes=1.00;No=0.00
29	Abnormal heart rhythm	regular=0;irregular=1
30	Symptom of psychological distress (based on loneliness, usefulness,and fearfulness)	Yes=1.00;No=0.00

Appendix 2. Definition of related covariates for CLHLS.

In CLHLS, the disease history of cardiovascular diseases and respiratory disease were collected by self-reported questions. Six activities were involved in ADL, such as dressing, bathing, using the toilet, continence, getting in/out of bed and chair and feeding. ADL impairment was defined if one of items indicates the inability to perform the task independently. Physical function were assessed by the abilities about upper extremities whether interviewee can put their “Hand behind neck”,“Hand behind lower back”,“Raise arms upright” with both hand, stand up from sitting in a chair, and pick up a book from the floor without help. Cognitive impairment was evaluated using the Mini-Mental State Examination (MMSE) in Appendix table. The total MMSE score ranges from 0 to 30 within 6 dimensions: orientation, registration, attention, language, memory, and visual construction skills.

Appendix table. Detailed Scoring Rubric for the Mini-mental State Examination (MMSE) score.

Items	Questions	Scoring rubric	Total score for each item
Orientation	What time of day is it right now (morning, afternoon, evening)?	0="wrong or unable answers"; 1="correct"	
	What is the month (Western or Chinese calendar) right now?	0="wrong or unable answers"; 1="correct"	
	What is the date (Chinese calendar day and month) of the mid-autumn festival?	0="wrong or unable answers"; 1="correct"	5
	What is the season right now, spring, summer, fall, winter?	0="wrong or unable answers"; 1="correct"	
Registration	What is the name of this district or town? (If the elderly is not a regular	0="wrong or unable answers"; 1="correct"	

	resident here, ask the elderly person to answer the name of the township or district where he originally lived)		
	I am now going to test your memory. I will mention three objects: table, apple, clothes (Mention these three objects without pausing:)	0="wrong or unable answers"; 1="correct"(1 point for each item, that is table, apple, clothes, a total of 3 points)	10
	Please repeat these three objects. Note: evaluation based on first attempt only. Number of repetitions. Note: fill in '0' if all three questions are answered correct at the first attempt; if answers are insufficient or incorrect on the first attempt, repeat the names of all objects until the interviewee is able to name all three of them (6 attempts at maximum). Write the number of attempts (e.g., '1', if all three objects are repeated correctly on first attempt). Write '7' if interviewee cannot repeat the names even after 6 attempts.	Score =7- Number of repetitions (range 0-7 points)	
Calculation	If I will ask you to spend 3 dollars from 20 dollars, then you must spend 3 dollars from the number you arrived at and continue to spend 3 dollars until you are asked to stop. Note: If the elderly forgets the previous number, interviewer can tell him or her the previous number for continue. But he or she is wrong this time even if the answer is correct. If he or she get right next round, then it counts right.	0="wrong or unable answers"; 1="correct" (The calculated 5 individual scores are independent of each other. The total score is 5 points.)	5
Copying figure	Ask the interviewee to draw the figure on B Card.	0="wrong" or "can't use pen to	

	Note: If all the sides and angles are correct and the figure in the middle is a quadrangle, then it counts right.	draw" or "not able to do this(disabled)"; 1="correct"	1
Word recall	Please repeat the three words (in any order) that I asked you to repeat a little while ago.<br>Please repeat the three words (in any order) that I asked you to repeat a little while ago.<br>table apple clothes<br>Note: No matter the order, just say the right name.	0="wrong or unable answers";<br>1="correct"<br>(1 point for each item, that is table, apple, clothes, a total of 3 points)	3
Comprehension	Point to the pen, then to the watch, and respectively ask the interviewed elderly: "What is this?"(1 point for each item, that is pen,<br>watch, a total of 2 points)	0="wrong or unable answers";<br>1="correct"	2
Repeating	I will now ask you to repeat the following sentence: What you plant, what you will get	0="wrong or unable answers";<br>1="correct"	1
Three-step tasks	The interviewer gives the elderly a piece of paper, and asked: Please take this paper in your right hand, fold it in half with both hands, and place the paper on the floor.<br>right hand<br>folding<br>on the floor<br>Note: Don't repeat requests and don't offer any favors. Notice if the action is correct.	0="wrong or unable answers";<br>1="correct"(1 point for each item, that is right<br>3<br>hand, folding, on the floor, a total	3

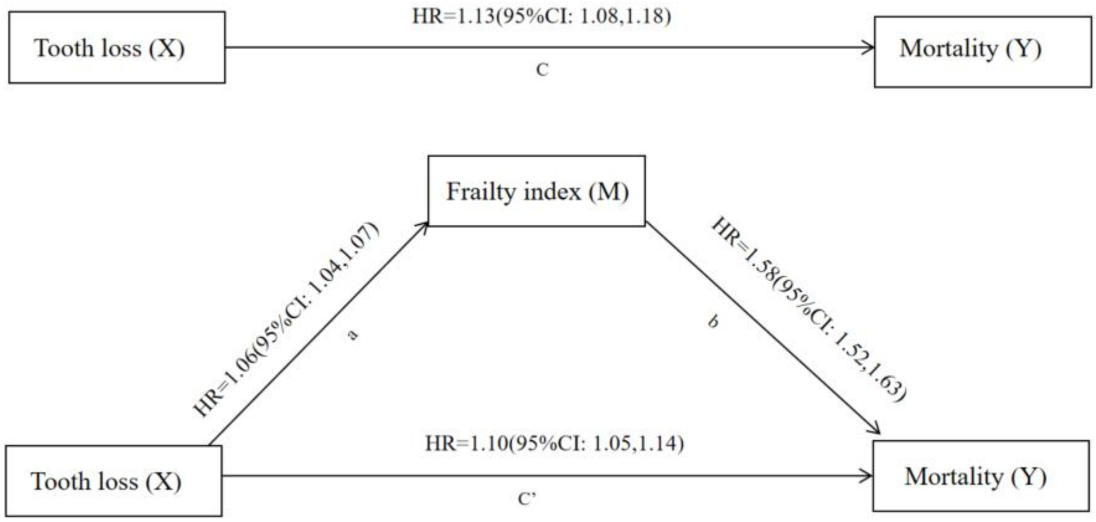

Appendix figure 1. Frailty index mediate the relationship between tooth loss and mortality after excluding missing data.

X: independent variable (cause); Y: dependent variable (outcome); M: mediator

Path a: the relationship between X and M

Path b: the relationship between M and Y, with X included in the model

Path c: the relationship between X and Y

Path c': the relationship between X and Y, with M included in the model

HR: odds ratio;

All models were adjusted for sex, age, education, marital status, smoking status, drinking status, dietary diversity and leisure activities included doing housework, reading, watching TV and listening to the radio, keeping pet and growing flowers.

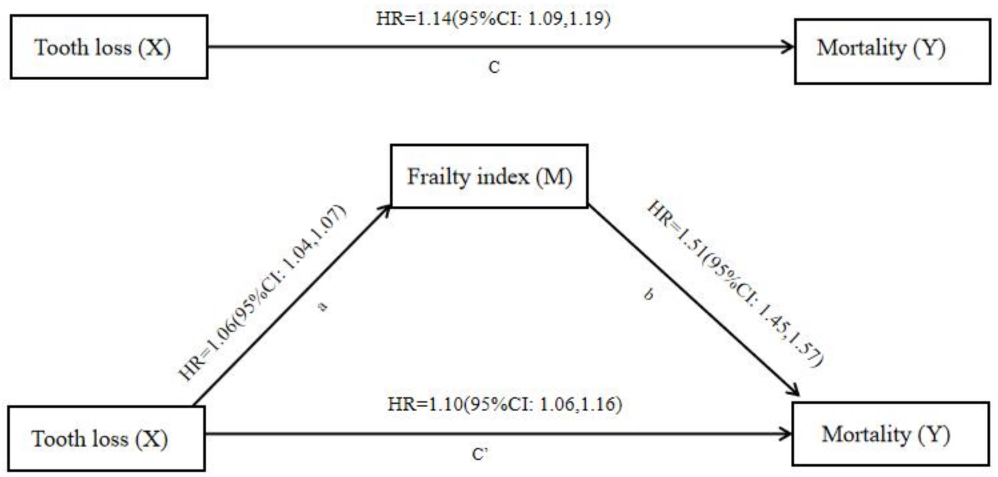

Appendix figure 2. Frailty index mediate the relationship between tooth loss and mortality after excluding mortality in the first 0.5 year..

X: independent variable (cause); Y: dependent variable (outcome); M: mediator
Path a: the relationship between X and M
Path b: the relationship between M and Y, with X included in the model
Path c: the relationship between X and Y
Path c': the relationship between X and Y, with M included in the model
HR: odds ratio;
All models were adjusted for sex, age, education, marital status, smoking status, drinking status, dietary diversity and leisure activities included doing housework, reading, watching TV and listening to the radio, keeping pet and growing flowers.

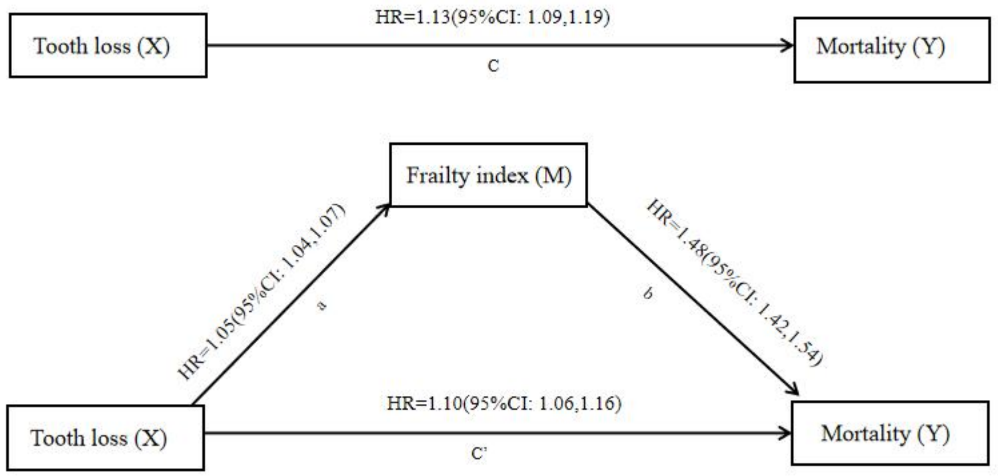

Appendix figure 3. Frailty index mediate the relationship between tooth loss and mortality after excluding mortality in the first 1 year.

X: independent variable (cause); Y: dependent variable (outcome); M: mediator

Path a: the relationship between X and M

Path b: the relationship between M and Y, with X included in the model

Path c: the relationship between X and Y

Path c': the relationship between X and Y, with M included in the model

HR: odds ratio;

All models were adjusted for sex, age, education, marital status, smoking status, drinking status, dietary diversity and leisure activities included doing housework, reading, watching TV and listening to the radio, keeping pet and growing flowers.

Appendix 3 STROBE Statement—checklist of items that should be included in reports of observational studies.

	Item No	Recommendation
**Title and abstract**	1	(*a*) Indicate the study's design with a commonly used term in the title or the abstract. **Page 1, line 1**
		(*b*) Provide in the abstract an informative and balanced summary of what was done and what was found. **Page 1 , line 19**
**Introduction**		
Background/rationale	2	Explain the scientific background and rationale for the investigation being reported. **Page 1, line 43**
Objectives	3	State specific objectives, including any prespecified hypotheses. **Page 2, line 133**
**Methods**		
Study design	4	Present key elements of study design early in the paper. **Page 2, line 139, and Appendix 1**
Setting	5	Describe the setting, locations, and relevant dates, including periods of recruitment, exposure, follow-up, and data collection. **Page 2, line 143, and Appendix 1**
Participants	6	(*a*) *Cohort study*—Give the eligibility criteria, and the sources and methods of selection of participants. Describe methods of follow-up. **Page 2, line 160, and Appendix 1**<br>*Case-control study*—Give the eligibility criteria, and the sources and methods of case ascertainment and control selection. Give the rationale for the choice of cases and controls<br>*Cross-sectional study*—Give the eligibility criteria, and the sources and methods of selection of participants
		(*b*) *Cohort study*—For matched studies, give matching criteria and number of exposed and unexposed. **Page 2, line 168, and Appendix 1**<br>*Case-control study*—For matched studies, give matching criteria and the number of controls per case
Variables	7	Clearly define all outcomes, exposures, predictors, potential confounders, and effect modifiers. Give diagnostic criteria, if applicable. **Page 2, line 176; Appendix table 1 and Appendix 2**
Data sources/ measurement	8*	For each variable of interest, give sources of data and details of methods of assessment (measurement). Describe comparability of assessment methods if there is more than one group. **Page 2, Page 3, Appendix table 1 and Appendix 2**
Bias	9	Describe any efforts to address potential sources of bias. **Page 3, line 381**
Study size	10	Explain how the study size was arrived at **Page 3, line 225**

Quantitative variables	11	Explain how quantitative variables were handled in the analyses. If applicable, describe which groupings were chosen and why. **Page 2, line 195, Appendix table 1 and Appendix 2**
Statistical methods	12	(*a*) Describe all statistical methods, including those used to control for confounding. **Page 3, line 270**
		(*b*) Describe any methods used to examine subgroups and interactions. **Page 4, line 454**
		(*c*) Explain how missing data were addressed. **Page 2, line 240**
		(*d*) *Cohort study*—If applicable, explain how loss to follow-up was addressed. **Page 3, line 272**<br>*Case-control study*—If applicable, explain how matching of cases and controls was addressed<br>*Cross-sectional study*—If applicable, describe analytical methods taking account of sampling strategy
		(*e*) Describe any sensitivity analyses. **Page 3, line 281**
**Results**		
Participants	13*	(a) Report numbers of individuals at each stage of study—eg numbers potentially eligible, examined for eligibility, confirmed eligible, included in the study, completing follow-up, and analysed **Page 3, line 422**
		(b) Give reasons for non-participation at each stage **Page 3, line 230**
		(c) Consider use of a flow diagram **Figure 1**
Descriptive data	14*	(a) Give characteristics of study participants (eg demographic, clinical, social) and information on exposures and potential confounders **Page 4, line 425**
		(b) Indicate number of participants with missing data for each variable of interest **Page 3, line 270**
		(c) *Cohort study*—Summarise follow-up time (eg, average and total amount). **Page 4, line 422**
Outcome data	15*	*Cohort study*—Report numbers of outcome events or summary measures over time. **Page 2, line 248**
		Case-control study—Report numbers in each exposure category, or summary measures of exposure
		Cross-sectional study—Report numbers of outcome events or summary measures
Main results	16	(*a*) Give unadjusted estimates and, if applicable, confounder-adjusted estimates and their precision (eg, 95% confidence interval). Make clear which confounders were adjusted for and why they were included. **Page 4, line 437**
		(*b*) Report category boundaries when continuous variables were categorized. **Table 1**
		(*c*) If relevant, consider translating estimates of relative risk into absolute risk for a meaningful time period.
Other analyses	17	Report other analyses done—eg analyses of subgroups and interactions, and sensitivity analyses. **Page 6, line 573**

Discussion

Key results	18	Summarise key results with reference to study objectives. **Page 6, line 616**
Limitations	19	Discuss limitations of the study, taking into account sources of potential bias or imprecision. Discuss both direction and magnitude of any potential bias. **Page 7, line 706**
Interpretation	20	Give a cautious overall interpretation of results considering objectives, limitations, multiplicity of analyses, results from similar studies, and other relevant evidence. **Page 7, line 622**
Generalisability	21	Discuss the generalisability (external validity) of the study results. **Page 7, line 669**
**Other information**		
Funding	22	Give the source of funding and the role of the funders for the present study and, if applicable, for the original study on which the present article is based. **Page 7, line 765**

*Give information separately for cases and controls in case-control studies and, if applicable, for exposed and unexposed groups in cohort and cross-sectional studies.
